# Patterns and management of chronic obstructive pulmonary disease in urban and rural China: a community-based survey of 25 000 adults across 10 regions

**DOI:** 10.1136/bmjresp-2017-000267

**Published:** 2018-02-19

**Authors:** Om P Kurmi, Kourtney J Davis, Kin Bong Hubert Lam, Yu Guo, Julien Vaucher, Derrick Bennett, Jenny Wang, Zheng Bian, Huaidong Du, Liming Li, Robert Clarke, Zhengming Chen

**Affiliations:** 1 Clinical Trial Service Unit and Epidemiological Studies Unit (CTSU), Nuffield Department of Population Health, University of Oxford, Oxford, UK; 2 Real World Evidence and Epidemiology, GlaxoSmithKline, Collegeville, Pennsylvania, USA; 3 National Co-ordinating Centre for China Kadoorie Biobank, Chinese Academy of Medical Sciences, Beijing, China; 4 Department of Epidemiology, Peking University Health Science Center, School of Public Health, Beijing, China

**Keywords:** respiratory, management, COPD, China

## Abstract

**Introduction:**

Chronic obstructive pulmonary disease (COPD) is the third leading cause of death worldwide, with COPD deaths in China accounting for one-third of all such deaths. However, there is limited available evidence on the management of COPD in China.

**Methods:**

A random sample of 25 011 participants in the China Kadoorie Biobank, aged 38–87 years, from 10 regions in China was surveyed in 2013–2014. Data were collected using interviewer-administered questionnaires on the diagnosis (‘doctor-diagnosed’ or ‘symptoms-based’) and management of COPD (including use of medication and other healthcare resources), awareness of diagnosis and severity of symptoms in COPD cases.

**Results:**

Overall, 6.3% of the study population were identified as COPD cases (doctor-diagnosed cases: 4.8% and symptom-based cases: 2.4%). The proportion having COPD was higher in men than in women (7.9% vs 5.3%) and varied by about threefold (3.7%–10.0%) across the 10 regions. Among those with COPD, 54% sought medical advice during the last 12 months, but <10% reported having received treatment for COPD. The rates of hospitalisation for COPD, use of oxygen therapy at home and influenza or pneumococcal vaccinations in the previous year were 15%, 3% and 4%, respectively. Of those with COPD, half had moderate or severe respiratory symptoms, and over 80% had limited understanding of their disease and need for treatment.

**Conclusion:**

Despite a high prevalence of COPD in China and its substantial impact on activities of daily living, knowledge about COPD and its management were limited.

**Key messages**

- Overall, about 6% of the study population had chronic obstructive pulmonary disease (COPD), but the prevalence varied almost threefold across the 10 study regions in China.
- Among COPD cases identified in China, one-half sought medical advice in the previous year and <1 in 10 reported having any treatment for COPD.
- Overall, four-fifths of COPD cases had limited understanding of their diagnosis or of the need for specific treatment for COPD.

## Introduction

Although there has been a gradual decline in age-standardised mortality rates worldwide over the last two decades, chronic obstructive pulmonary disease (COPD) is still a leading cause of death,^1^ accounting for about 3 million deaths in 2010.^1^ About 70% of all COPD deaths worldwide now occur in South and East Asia,^2^ where the burden of COPD is projected to increase over the next few decades due to a high prevalence of smoking and rapidly ageing populations.^3 4^ China alone contributed to about one-third (0.9 million/year) of all deaths from COPD worldwide. Within China, the prevalence of COPD varies substantially by region^5 6^ and death rates attributed to COPD are twofold greater in South-West^5^compared with North-East regions for reasons that are not fully understood.^3^

Despite the high burden of COPD, there is limited available evidence on diagnosis and management of COPD in China. Using data on 25 000 adults from a nationwide survey in 2013–2014 across 10 regions of China, as part of the China Kadoorie Biobank (CKB), we examined the diagnosis of COPD and the knowledge, management and severity among cases with COPD, overall and by age, sex and region.

## Methods

### Study population

**Figure 1:**
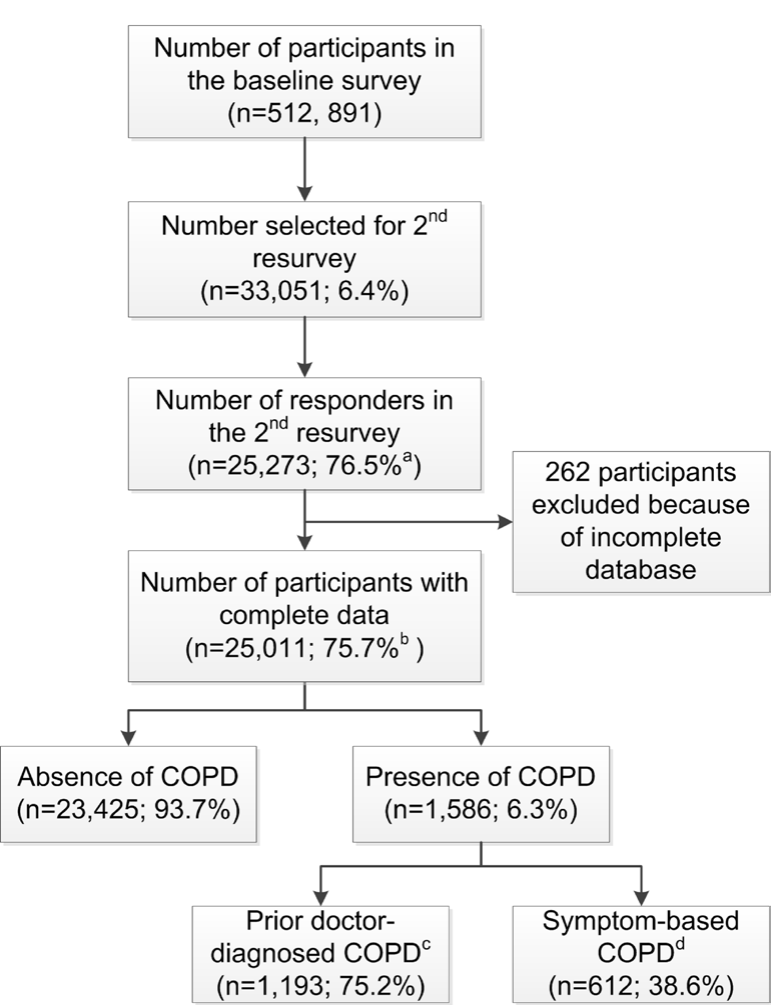
Flow diagram of participants included in the survey. ^a^Response rate. ^b^Actual response rate (participants included in the analysis and had completed all sections that comprised questionnaire, physical measurements and tests during the second resurvey). ^c^Chronic obstructive pulmonary disease (COPD) defined by history of doctor diagnosis of COPD=COPD and/or chronic bronchitis and/or emphysema. ^d^Symptom-based COPD are those with self-reported chronic cough, that is, productive cough for at least 3 months for 2 consecutive years.

The CKB study is a prospective study of over 0.5 million adults, aged 30–79 years at baseline, who were recruited from 10 geographically diverse regions across China between 2004 and 2008.^7 8^ A random sample of about 6% surviving participants underwent periodic resurveys every 4–5 years including repeat interviews, physical measurements and collection of biological samples. The present report is based on the 25 011 individuals who participated in the second resurvey during 2013–2014, and had an overall response rate of 76% (figure 1). Detailed information was collected using interviewer-administered laptop-based questionnaires on personal and sociodemographic characteristics, lifestyle (including questions on diet, smoking, alcohol and physical activity) and medical history (including questions on specific conditions, such as COPD). Each individual provided a written consent to participate in this study.

### Definition of COPD cases

In this report, COPD cases (see online supplementary figure 1) were defined using two different criteria:

- Participants who answered ‘yes’ to the question "Have you had chronic productive cough for 3 months or more during at least two consecutive years" were classified as having *symptom-based COPD*^9^;
- Participants who answered ‘yes’ to the question "Has a doctor ever told you that you had COPD, chronic bronchitis or emphysema" were classified as having *doctor-diagnosed COPD*.

10.1136/bmjresp-2017-000267.supp1 Supplementary data

### Assessment of severity and management of COPD

All participants with COPD were requested to complete additional questions on: (i) COPD-related health status using the COPD Assessment Test (CAT)^10^ and (ii) knowledge, severity and management of COPD, including visits to health professionals, regular use of medication within the previous 7 days, management of exacerbations, influenza and pneumococcal vaccinations and use of oxygen therapy in the last 12 months (see online supplementary appendix 1).^11^

Participants with doctor-diagnosed COPD were also asked to provide: (i) age at first diagnosis; (ii) use of treatment at the time of the resurvey; (iii) any hospitalisation for an acute exacerbation of COPD and (iv) date of last hospitalisation for COPD.

### Lung function measurement

All participants except those with contraindications^12^ had lung function assessed using a personal computer-based spirometer (Pneumotrac 6800, Vitalograph, Buckingham, UK) by trained technicians. After conducting some practice exhalations, the results of at least two reproducible measurements (difference of at least two forced expiratory volume in 1 s and forced vital capacity <150 mL) were recorded. The spirograms of those with symptom-based or doctor-diagnosed COPD were reviewed independently for quality by two authors (OK and KBHL) following the European Respiratory Society/American Thoracic Society (ERS/ATS) guidelines.^13^ Out of 1586 doctor-diagnosed/symptom-based COPD participants, 55 (3.5%) did not have lung function tests conducted due to contraindications, 1282 (83.7%) of those with lung function tests had acceptable spirometry and 600 (37.8%) had COPD confirmed by spirometry (defined by lower limit of normal criteria) based on prebronchodilator measured lung function.^14^

### Statistical analysis

Linear and logistic regression analyses were used for continuous and binary outcomes, respectively, after stratification for age (five groups), sex and region (10 groups). Likewise, adjusted means (SD) or proportions with their associated 95% CI, respectively, were also stratified by age, sex and region at the time of second resurvey. Similar analyses were carried out to estimate age (five groups) and sex-adjusted means (and their SD) or proportions, respectively, for patient-reported symptoms and use of medication by region. All the above analyses were repeated after stratification for the three classification schemes for COPD: symptom-based, doctor-diagnosed and those spirometry-confirmed.

## Results

### Characteristics of participants with COPD

**Figure 2:**
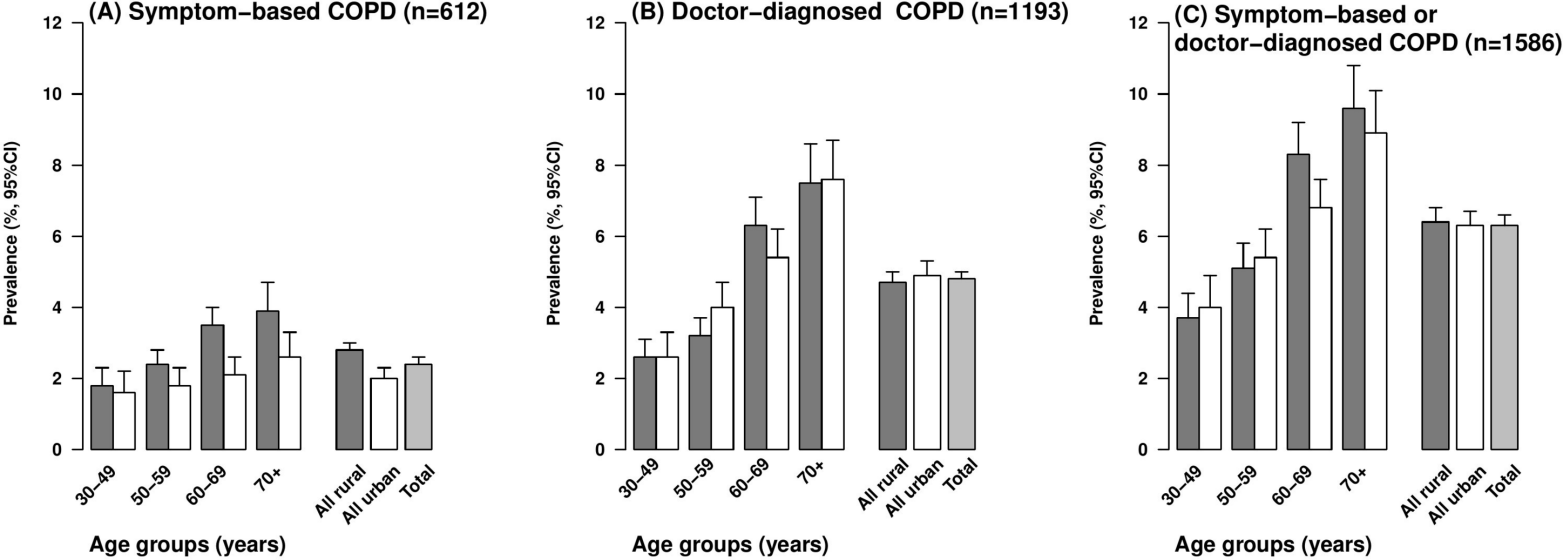
Prevalence of chronic obstructive pulmonary disease (COPD) defined by different criteria by age, and place of residence. The dark grey bar represents ‘rural’ participants and white bar represents ‘urban’ participants.

**Figure 3:**
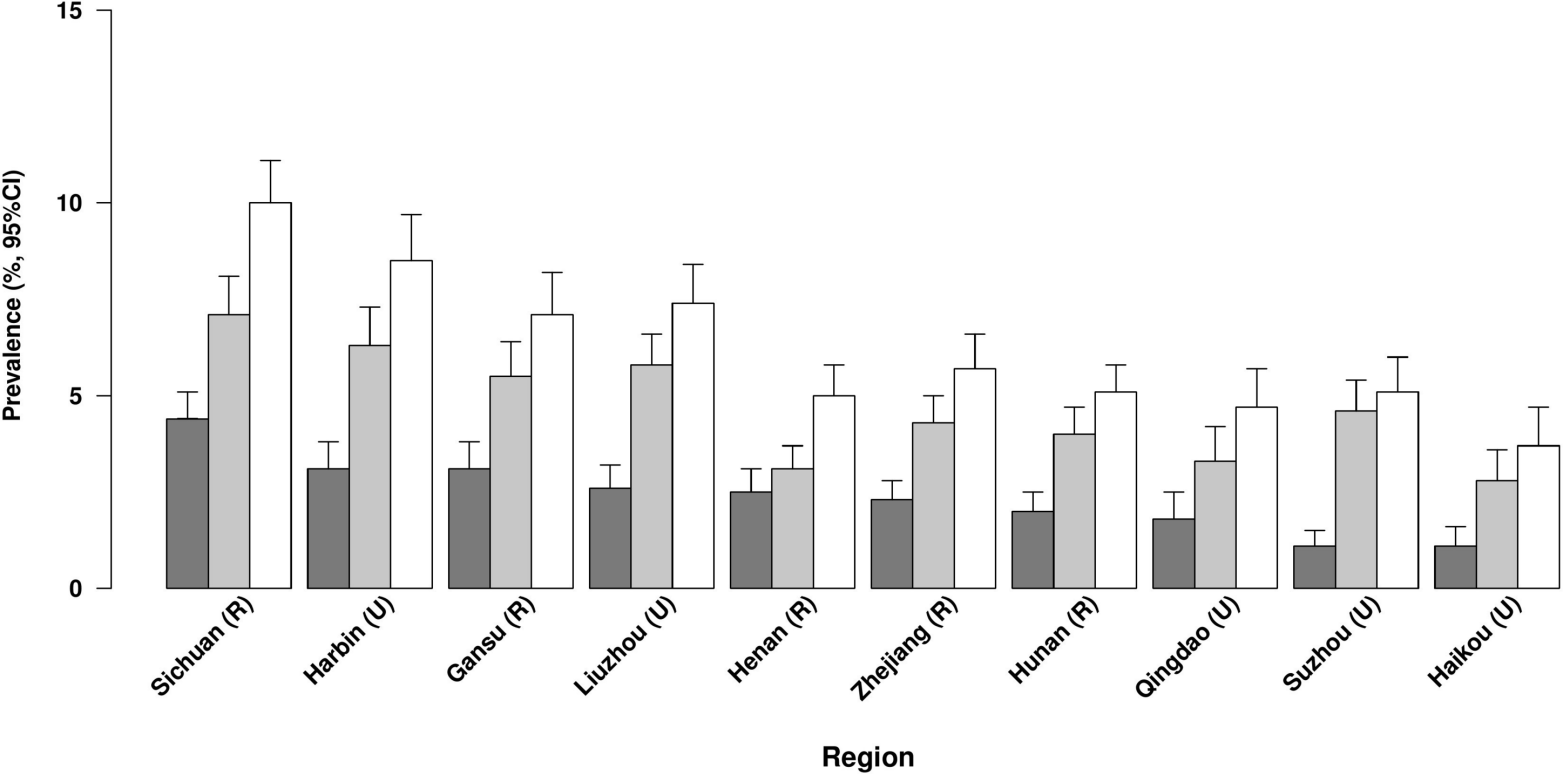
Prevalence of chronic obstructive pulmonary disease (COPD) defined by different criteria by region. The dark grey bar represents ‘symptom-based COPD’, grey bar represents ‘doctor-diagnosed COPD’ and the white bar represents ‘symptom-based or doctor-diagnosed COPD’ participants.

Overall, 6.3% of participants had COPD, with two-thirds of the cases (4.8%) being doctor-diagnosed and one-third (2.4%) being symptom-based (figure 1). The proportion having COPD increased with age (from 3.8% at age 30–49 years to 9.3% at age ≥70 years) and was 50% greater in men than in women (7.9% vs 5.3%) (table 1 and figure 2). Men reported a higher proportion of symptom-based COPD than women (58% vs 42%). The gender differences in COPD reflect the much higher prevalence of smoking in men than in women (73% vs 4%), but the combined ex-smokers had the highest prevalence of COPD (12.8%) (table 1). Overall, the mean age of ex-smokers (63.8 years) was about 5 years older than that for either current smokers (58.4 years) or never regular smokers (58.7 years). Although the overall proportion having COPD was similar in rural (6.5%) and urban (6.2%) areas, it varied by about threefold across the geographically defined 10 regions (from 3.7% in Haikou to 10.0% in Sichuan; figure 3). Although, the proportion of participants with COPD was slightly lower in never regular smokers compared with the overall participants, this proportion in never-smokers varied threefold between regions (2.5% in Haikou vs 8.5% in Sichuan).

**Table 1:** Prevalence of symptom-based COPD, doctor-diagnosed COPD or either, by second resurvey characteristics

Characteristics	Study population	Symptom-based COPD* % (95% CI)	Doctor-diagnosed COPD† % (95% CI)	Symptom-based or doctor-diagnosed COPD‡ % (95% CI)
All participants (%)	25 011	612 (2.4)	1193 (4.8)	1586 (6.4)
Age at resurvey (years)				
30–49	5500	1.7 (1.4 to 2.1)	2.6 (2.2 to 3.0)	3.8 (3.3 to 4.4)
50–59	7722	2.1 (1.8 to 2.5)	3.6 (3.2 to 4.0)	5.2 (4.7 to 5.7)
60–69	7449	2.9 (2.5 to 3.3)	6.0 (5.4 to 6.5)	7.7 (7.0 to 8.3)
70+	4340	3.3 (2.8 to 3.9)	7.5 (6.7 to 8.3)	9.3 (8.4 to 10.2)
Sex				
Men	9580	3.7 (3.3 to 4.0)	5.4 (5.0 to 5.9)	7.9 (7.4 to 8.5)
Women	15 431	1.7 (1.5 to 1.9)	4.3 (4.0 to 4.6)	5.3 (5.0 to 5.7)
Area				
Rural	14 235	2.8 (2.5 to 3.1)	4.7 (4.4 to 5.1)	6.5 (6.1 to 6.9)
Urban	10 776	2.0 (1.7 to 2.3)	4.8 (4.4 to 5.2)	6.2 (5.7 to 6.6)
Education				
None	5068	2.3 (1.2 to 3.4)	4.0 (3.1 to 4.9)	5.7 (4.2 to 7.1)
Primary	8032	2.2 (1.9 to 2.5)	4.5 (4.0 to 5.0)	5.9 (5.3 to 6.4)
Secondary or tertiary	11 911	2.5 (2.1 to 3.0)	4.7 (4.1 to 5.3)	6.6 (5.9 to 7.3)
Household income (¥)				
<10 000	2175	2.9 (2.2 to 3.5)	5.5 (4.2 to 6.7)	7.3 (6.0 to 8.7)
10 000–19 999	3079	3.1 (2.1 to 4.0)	4.8 (3.6 to 6.0)	6.7 (5.3 to 8.0)
≥20 000	19 757	2.4 (2.1 to 2.6)	4.8 (4.5 to 5.1)	6.3 (6.0 to 6.7)
Smoking status				
Never	18 274	1.8 (1.6 to 2.0)	4.6 (4.2 to 4.9)	5.7 (5.3 to 6.1)
Ex-regular	1611	3.6 (1.9 to 5.4)	11.1 (9.3 to 12.9)	12.8 (10.6 to 15.0)
Current regular	5126	2.9 (2.4 to 3.4)	3.7 (2.6 to 4.8)	5.9 (4.8 to 7.0)
Body mass index (kg/m²)				
<18.5	950	4.0 (2.7 to 5.2)	8.9 (7.0 to 10.8)	10.7 (8.6 to 12.7)
18.5–25.0	14 289	2.3 (2.0 to 2.5)	4.5 (4.1 to 4.8)	5.9 (5.5 to 6.3)
≥25.0	9772	2.5 (2.2 to 2.9)	4.9 (4.4 to 5.4)	6.7 (6.2 to 7.2)
Physical activities (MET-hour/day)				
<10	14 129	2.7 (2.3 to 3.1)	4.9 (4.5 to 5.3)	6.6 (6.1 to 7.2)
10–14	1722	2.3 (1.6 to 3.1)	4.3 (3.2 to 5.3)	5.6 (4.4 to 6.8)
≥14	9160	2.3 (1.8 to 2.7)	4.8 (4.1 to 5.6)	6.3 (5.5 to 7.1)
Self-assessed health status				
Good/excellent	10 943	1.5 (1.3 to 1.7)	2.7 (2.4 to 3.0)	3.8 (3.5 to 4.2)
Fair	10 851	2.4 (2.1 to 2.7)	4.9 (4.5 to 5.4)	6.7 (6.2 to 7.1)
Poor	3217	5.4 (4.6 to 6.2)	10.0 (8.9 to 11.1)	12.9 (11.7 to 14.1)

*Symptom-based COPD are those with self-reported chronic cough, that is, productive cough for at least 3 months for two consecutive years.

†COPD defined by history of doctor diagnosis of COPD, where COPD=COPD and/or chronic bronchitis and/or emphysema.

‡Either * or †; standardised for region, age group and gender as appropriate.

COPD, chronic obstructive pulmonary disease; MET, metabolic equivalent of task.

Doctor-diagnosed COPD was more common than symptom-based COPD in older participants (≥60 years) (figure 2). Irrespective of the case definition, participants reporting COPD tended to have a lower income, be underweight (BMI <18.5 kg/m^2^) and have poor self-assessed health status (table 1). Concomitant illnesses including asthma, ischaemic heart disease, rheumatoid arthritis and tuberculosis were usually twofold more frequent among those with doctor-diagnosed COPD compared with those with symptom-based COPD (see online supplementary table 1).

### Management of COPD

Just over half of those with COPD visited a health professional in the previous 12 months (prior to the survey), slightly higher among those with doctor-diagnosed COPD compared with symptom-based COPD (63% vs 43%); the majority of which (34%) involved consultation with a general physician (table 2). Compared with their urban counterparts, rural residents were less likely to seek medical advice (50% vs 61%) despite a higher proportion of them reporting more severe conditions (21% vs 15%) (see online supplementary table 2).

**Table 2:** Disease severity and management measures, by COPD subtypes

Characteristics	Study population	Symptom-based COPD % (95% CI)	Doctor-diagnosed COPD % (95% CI)	Symptom-based or doctor-diagnosed COPD % (95% CI)
Participants	1586	612	1193	1586
Lung function (mean±SD)				
FEV_1_ (L)	1.8±0.1	1.9±0.2	1.7±0.1	1.8±0.1
FVC (L)	2.6±0.2	2.7±0.2	2.5±0.2	2.6±0.2
FEV_1_/FVC (%)	67.1±2.5	68.3±2.4	65.6±2.6	67.1±2.5
CAT score				
<10	882	50.1 (48.7 to 51.5)	54.2 (53.2 to 55.2)	55.6 (54.8 to 56.5)
10–20	514	32.9 (31.6 to 34.2)	33.0 (32.0 to 33.9)	32.4 (31.6 to 33.2)
>20	190	17.0 (15.9 to 18.1)	12.8 (12.2 to 13.5)	12.0 (11.4 to 12.5)
Awareness of condition and treatment				
Well informed	295	13.8 (12.9 to 14.8)	20.7 (19.9 to 21.5)	18.6 (17.9 to 19.3)
Inadequately/poorly informed	1114	72.2 (71.0 to 73.5)	69.8 (68.9 to 70.7)	70.2 (69.5 to 71.0)
Do not know	177	13.9 (13.0 to 14.9)	9.4 (8.9 to 10.0)	11.2 (10.6 to 11.7)
Self-reported severity of chronic lung disease				
Mild	713	44.0 (42.6 to 45.3)	41.1 (40.1 to 42.1)	45.0 (44.1 to 45.8)
Moderate	578	35.0 (33.7 to 36.4)	38.1 (37.1 to 39.1)	36.4 (35.6 to 37.3)
Severe	295	21.0 (19.8 to 22.1)	20.8 (20.0 to 21.6)	18.6 (17.9 to 19.3)
Vaccination (last 12 months)				
No	1527	95.8 (95.3 to 96.4)	96.0 (95.6 to 96.4)	96.3 (96.0 to 96.6)
Yes	59	4.2 (3.6 to 4.7)	4.0 (3.6 to 4.4)	3.7 (3.4 to 4.0)
Influenza vaccination	54	3.5 (3.0 to 4.0)	3.8 (3.4 to 4.2)	3.4 (3.1 to 3.7)
Pneumococcal vaccination	14	1.4 (1.1 to 1.8)	0.9 (0.7 to 1.1)	0.9 (0.7 to 1.0)
Healthcare professional consultations (last 12 months)				
No	721	56.7 (55.3 to 58.1)	37.0 (36.0 to 37.9)	45.5 (44.6 to 46.3)
Yes	865	43.3 (41.9 to 44.7)	63.0 (62.1 to 64.0)	54.5 (53.7 to 55.4)
General physician	536	27.9 (26.6 to 29.1)	38.9 (38.0 to 39.9)	33.8 (33.0 to 34.6)
Respiratory specialist	305	13.7 (12.7 to 14.7)	23.2 (22.4 to 24.1)	19.2 (18.6 to 19.9)
Cardiologist/heart specialist	74	3.9 (3.3 to 4.4)	5.3 (4.9 to 5.8)	4.7 (4.3 to 5.0)
Traditional Chinese medicine doctor	132	7.2 (6.5 to 7.9)	9.7 (9.1 to 10.3)	8.3 (7.9 to 8.8)
Other medical professional	104	5.7 (5.0 to 6.3)	7.3 (6.8 to 7.8)	6.6 (6.1 to 7.0)
Hospitalisation for COPD (last 12 months)				
None	1333	87.4 (86.5 to 88.3)	81.3 (80.5 to 82.1)	84.7 (84.1 to 85.4)
One	182	8.6 (7.8 to 9.4)	14.2 (13.5 to 14.9)	11.6 (11.0 to 12.1)
≥Two	58	3.9 (3.4 to 4.5)	4.5 (4.1 to 5.0)	3.7 (3.4 to 4.0)
Oxygen therapy at home (last 12 months)				
No	1523	96.7 (96.1 to 97.2)	96.5 (96.1 to 96.9)	97.2 (96.9 to 97.5)
Yes	44	3.3 (2.8 to 3.9)	3.5 (3.1 to 3.9)	2.8 (2.5 to 3.1)
Medication for COPD exacerbation (last 12 months)				
No	1072	71.8 (70.6 to 73.1)	62.4 (61.5 to 63.4)	67.6 (66.8 to 68.4)
Yes	514	28.2 (26.9 to 29.4)	37.6 (36.6 to 38.5)	32.4 (31.6 to 33.2)
Antibiotics	483	26.5 (25.3 to 27.7)	35.5 (34.6 to 36.4)	30.5 (29.7 to 31.2)
Oral steroids	107	6.1 (5.4 to 6.8)	7.9 (7.4 to 8.4)	6.7 (6.3 to 7.2)
Injectable steroids	70	3.0 (2.5 to 3.4)	5.4 (5.0 to 5.9)	4.4 (4.1 to 4.8)
Number of COPD exacerbations after medication				
None	1185	77.4 (76.3 to 78.6)	70.4 (69.5 to 71.3)	74.7 (74.0 to 75.4)
One	202	8.4 (7.6 to 9.2)	15.5 (14.8 to 16.2)	12.7 (12.2 to 13.3)
≥Two	199	14.2 (13.2 to 15.2)	14.1 (13.4 to 14.8)	12.5 (12.0 to 13.1)
Prescribed medication (last 7 days)				
No	1447	90.8 (89.9 to 91.6)	89.8 (89.2 to 90.4)	91.2 (90.7 to 91.7)
Yes	139	9.2 (8.4 to 10.1)	10.2 (9.6 to 10.8)	8.8 (8.3 to 9.3)
Short-acting bronchodilators	25	2.1 (1.7 to 2.5)	2.0 (1.7 to 2.2)	1.6 (1.4 to 1.8)
Long-acting bronchodilators	16	1.5 (1.2 to 1.9)	1.2 (1.0 to 1.4)	1.0 (0.8 to 1.2)
Oral bronchodilators	19	1.0 (0.7 to 1.3)	1.4 (1.2 to 1.7)	1.2 (1.0 to 1.4)
Any inhalers	11	0.5 (0.3 to 0.7)	0.8 (0.6 to 0.9)	0.7 (0.6 to 0.8)
Traditional Chinese medicine	33	2.1 (1.6 to 2.5)	2.6 (2.3 to 2.9)	2.1 (1.8 to 2.3)
Other treatments	94	6.4 (5.7 to 7.1)	6.7 (6.2 to 7.2)	5.9 (5.5 to 6.3)

*All data standardised for region, age group and gender as appropriate. Short-acting bronchodilators (short-acting beta_2_-agonist/short-acting muscarinic antagonist); long-acting bronchodilators (long-acting beta_2_-agonist/long-acting muscarinic antagonist).

CAT, COPD Assessment Test; COPD, chronic obstructive pulmonary disease; FEV_1_, forced expiratory volume in 1 s; FVC, forced vital capacity.

**Figure 4:**
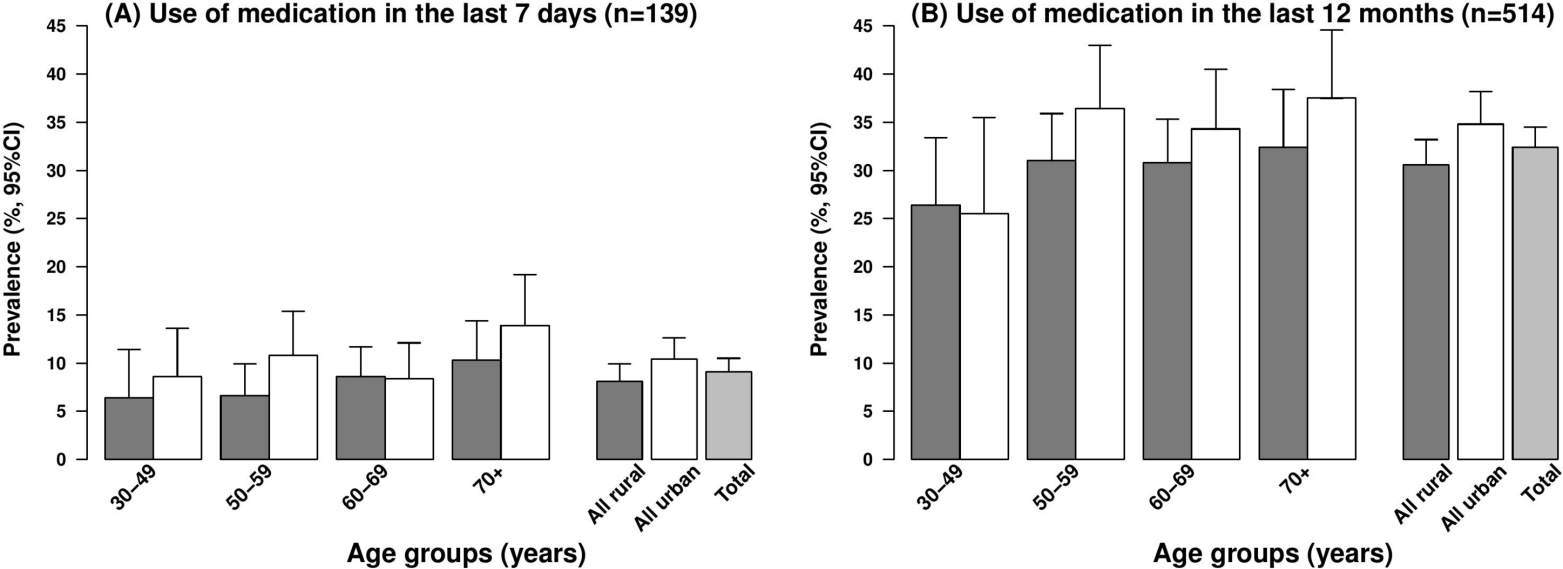
Use of medication among chronic obstructive pulmonary disease (COPD) cases by age and place of residence among 1586 COPD cases. The dark grey bar represents ‘rural’ and the white bar represents ‘urban’ participants. Medications include antibiotics or oral steroids or injectable steroids.

Use of any medication in the last 7 days to alleviate symptoms and to reduce the frequency and severity of exacerbations was reported by 9% of the COPD cases, with short-acting bronchodilators reported as the most frequently used medication. Use of oxygen therapy at home was low (2.8%), as were guideline recommended influenza or pneumococcal vaccinations (3.7%) (table 2). Doctor-diagnosed COPD cases were more likely to have used prescribed medications in the last 7 days than symptom-based COPD cases (10.2% vs 9.2%). The use of medication was greater in older age groups in both rural and urban areas (figure 4). Interestingly, Sichuan, the region with the highest prevalence of COPD, had the lowest reported use of medication (10.3%) in the last 12 months (table 3). Overall, 15% of cases were ever hospitalised for their pulmonary condition during the last 12 months and about one-third reported having received antibiotics, or oral or injectable corticosteroids for an acute COPD exacerbation. Around 25% of those who were recently prescribed medication reported having at least one COPD exacerbation in the last 12 months (table 2).

**Table 3:** Use of COPD medication among COPD cases, in the last 7 days or the last 12 months prior to resurvey by gender, region, socioeconomic and smoking status

Characteristics	Study population	Use of medication* (%, 95% CI) Last 7 days	Use of medication† (%, 95% CI) Last 12 months
Participants	1586	139	514
Sex			
Men	772	9.6 (8.6 to 10.7)	30.0 (28.5 to 31.6)
Women	814	8.0 (7.0 to 8.9)	34.6 (33.0 to 36.2)
Area			
Rural (R)	911	7.8 (6.9 to 8.7)	30.6 (29.1 to 32.1)
Urban (U)	675	10.1 (9.0 to 11.2)	34.7 (32.9 to 36.5)
Regions‡			
Sichuan (R)	265	7.0 (5.5 to 8.5)	10.3 (8.5 to 12.2)
Harbin (U)	183	10.8 (8.6 to 13.0)	31.7 (28.3 to 35.1)
Gansu (R)	173	10.4 (8.1 to 12.8)	65.7 (62.1 to 69.3)
Liuzhou (U)	216	10.5 (8.5 to 12.6)	26.2 (23.3 to 29.2)
Henan (R)	147	0.6 (0.0 to 1.2)	14.9 (11.9 to 18.0)
Zhejiang (R)	174	7.0 (5.0 to 8.9)	22.8 (19.7 to 25.9)
Hunan (R)	152	14.4 (11.4 to 17.3)	49.8 (45.7 to 54.0)
Qingdao (U)	81	10.5 (7.2 to 13.8)	29.4 (24.4 to 34.4)
Suzhou (U)	145	10.0 (7.7 to 12.4)	51.5 (47.4 to 55.5)
Haikou (U)	50	7.5 (4.1 to 11.0)	40.9 (33.9 to 47.9)
Household income (¥)			
<10 000	188	9.6 (7.7 to 11.4)	35.1 (32.7 to 37.5)
10 000–19 999	208	9.1 (7.6 to 10.6)	36.2 (33.8 to 38.6)
≥20 000	1190	8.7 (8.2 to 9.3)	31.9 (31.0 to 32.8)
Education			
None	326	8.5 (7.1 to 9.8)	31.1 (28.6 to 33.6)
Primary	530	9.3 (8.3 to 10.3)	36.8 (35.4 to 38.3)
Secondary or tertiary	730	8.2 (7.4 to 8.9)	28.3 (27.1 to 29.5)
Smoking status			
Never	984	9.0 (8.2 to 9.7)	32.0 (30.9 to 33.0)
Ex-regular	177	17.0 (14.6 to 19.4)	39.4 (36.5 to 42.3)
Current regular	425	8.3 (6.6 to 10.0)	27.1 (24.9 to 29.4)

*Medication questionnaire was completed only by those who had symptom-based COPD or history of doctor diagnosed COPD in the second resurveys; medication in last 7 days (short-acting bronchodilators, long-acting bronchodilators, oral bronchodilators, any inhaled medications, traditional Chinese medicine and any other treatments prescribed for COPD).

†Medication in last 12 months (antibiotics, oral corticosteroids, injectable corticosteroids). Standardised for region, age group and gender as appropriate.

‡The regions are ordered by COPD prevalence as in figure 3.

COPD, chronic obstructive pulmonary disease.

### Severity of COPD, functional status and awareness of diagnosis

Overall, one-fifth of the participants with COPD reported having either severe or very severe respiratory symptoms, with a slightly higher proportion in never-smokers (20%) and rural dwellers (21%) compared with ever-smokers (18%) and urban dwellers (15%), respectively (table 2, online supplementary tables 2 and 3). A moderate or severe CAT score (≥10) was reported by 44% of COPD cases. Doctor-diagnosed COPD cases had better functional status despite having slightly lower levels of lung function indices than symptom-based COPD cases (table 2).

Over 80% of COPD cases reported that they were less than adequately or poorly informed about their condition and its treatment. The low levels of disease awareness were more pronounced in rural (87%) than in urban areas (73%) (see online supplementary table 2). Participants with doctor-diagnosed COPD were 1.5-times more likely to be aware of their condition compared with those with only symptom-based COPD (21% vs 14%; table 2).

## Sensitivity analyses

Among the 1586 COPD cases (by either definitions), 600 (38%) were confirmed by spirometry using the lower limit of normal criteria (see online supplementary table 4). Those who had spirometric evidence of airflow obstruction in addition to having symptoms or a physician diagnosis were more likely to be older, males, and live in rural areas (see online supplementary table 5). As expected, they had a more severe condition, as noted by the higher proportions of CAT score ≥10, self-reported severity and hospitalisation due to COPD (see online supplementary table 4), compared with all self-reported COPD cases (table 2). Nevertheless, there was no material difference in terms of the awareness and management of the disease between the spirometry-confirmed subset and the combined group of symptom-based or physician diagnosed individuals.

## Discussion

The present cross-sectional survey of 25 011 Chinese adults conducted in the China Kadoorie Biobank cohort in 2013–2014 demonstrated substantial variation in COPD prevalence by age, sex and region. It also highlighted low levels of understanding about their diagnosis, unmet medical needs (indicated by symptoms, exacerbations and COPD-related health status) and very low use of appropriate medication for COPD in China. Indeed, use of medication was lowest in the rural regions where COPD was more common. These findings highlight the need for better recognition of COPD as a major healthcare problem in China and the need to improve the global management of this disease in the general population.

The prevalence of COPD was 6.3% in the present study and was similar to previous estimates reported for equivalent aged populations in China: 6.5% by the Regional COPD working group in 2003,^15^ 2%–8% by the more recent Global Burden of Diseases Study^16^ and 8.2% for those ≥40 years based on postbronchodilator spirometry from a large survey comprising seven rural and urban sites in China.^6^ Using the same definition as in the present study, the Continuing to Confront COPD International Survey (C2C),^17^ a population-based survey estimated that the prevalence of COPD ranged from 6.5% to 11.8% in 12 countries among representative samples of adults aged 40+ years, the samples being selected through telephone and face-to-face interviews from general population. While there were no data from China in C2C, the estimates for COPD were 7.0% in Japan and 8.2% in South Korea. Few studies have investigated the reasons for variations in the pattern of COPD between or within countries. The present study demonstrated substantial region-specific differences (even within rural and urban areas), irrespective of smoking status and age groups. Access and utilisation of the healthcare services and, in the Chinese context, the capacity of healthcare professionals to diagnose COPD, could account for some of the observed differences across regions.^18 19^ However, further research in addition to the risk associated with tobacco exposure is needed to examine the role of environmental risk factors such as nutritional status, infection in early life, air pollution and occupational exposures for risk of COPD, and the quality of local healthcare provision which could further contribute to the regional variation observed.

In the present study, only 9% of the COPD cases (approximately 12% in those who were also diagnosed by spirometry) reported receiving treatment for their disease, which was comparable to South Korea (11%), but markedly lower than estimates for Russia (21%) or for the UK (72%).^17^ A higher rate of treatment among doctor-diagnosed and urban cases in the present study may reflect the fact that these COPD cases had more access to higher quality medical care.^20^ Short-acting bronchodilators were the most frequently used medication in the present study in contrast to the international recommendations that advocate the use of long-acting bronchodilators (either beta_2_-agonists or antimuscarinics) to reduce the symptoms and the frequency of exacerbations of COPD.^21–23^ As COPD-related symptoms, assessed by either the CAT or the Modified British Medical Research Council Questionnaire, should guide drug treatment according to Global Initiative for Chronic Obstructive Lung Disease (GOLD) guidelines, at least 44% of the COPD cases with a CAT scores ≥10 would be eligible to receive long-acting bronchodilators in the present study.^23^ While it is well established that influenza immunisation is a highly effective measure to reduce rates of exacerbations and hospitalisations due to COPD,^24 25^ the reported use of vaccination was low, perhaps reflecting absence of a routine influenza vaccination programme in China.^26^

Exacerbations of COPD were reported more frequently in urban than in rural areas. This may reflect a more crowded living environment, higher levels of ambient air pollution or a greater awareness of the link between pollution and exacerbation of COPD in the cities.^27–30^ Alternatively, cases living in rural regions may have been less likely to seek medical advice for the alleviation of symptoms due to poor access to healthcare or due to financial constraints.^31 32^ The annual hospitalisation rate (15%) for COPD observed in this survey was comparable to the global average (15%) reported from 12 countries by Landis *et al.*^17^ Despite the lower rate of exacerbations of COPD reported in rural areas in China, hospitalisation rates indicating severe exacerbations were similar in rural and urban areas. Exacerbation seemed to be a factor associated with receiving some form of medical treatment as 78% of those with an exacerbation during the previous 12 months reported receiving treatment for their COPD, consistent with standard recommendations.^23^

Participants with COPD had poor awareness of their diagnosis, and awareness was higher for the doctor-diagnosed than for symptom-based COPD cases. Cases living in urban areas were better informed than their rural counterparts, perhaps due to better socio-educational status or better access to healthcare services. In contrast, in rural areas the disease was more frequently described as severe or very severe, consistent with previous reports elsewhere.^33–35^ Variation by case definition, rural/urban or smoking status was not observed in the CAT score measuring COPD-related health status, although there was variation in CAT across regions. Similarly limited utility of the CAT in the characterisation of COPD has also been previously reported in China.^18^

The present study has several strengths, including the large number of COPD cases, the variation in geographic region and levels of socioeconomic circumstances included in the study population and clinical measures such as spirometry testing along with self-reported information. As the survey was conducted in 2013–2014, the findings are likely to reflect the current management of COPD in China. The present study also has a number of limitations. Although the response rate was reasonable for the second resurvey that is the basis of the current manuscript, the baseline response rate was only about 30%, so results may not be representative of the general population. The COPD cases were defined through self-reported questionnaires and prebronchodilator spirometry (postbronchodilator is the current gold standard for diagnosis of COPD).^23^ Nevertheless, the estimates of COPD prevalence in the present study are consistent with previous studies in China^6 15 16^ and are comparable in magnitude with those reported in other countries using a similar approach. However, we cannot completely exclude the possibility that some of the cases with reported COPD may also have asthma (asthma-COPD overlap syndrome).^17^ We would also argue that a symptom-based and self-reported definition is better suited for the aim of this study, which focused on the management and awareness of COPD as spirometry is not widely used in low-income and middle-income countries. Indeed, even when restricting to cases that were confirmed by spirometry, which tended to be more severe, the overall message that COPD has not been adequately managed was unaltered. Likewise, we relied on self-reported healthcare and medication use, which might have underestimated the true prevalence. Nevertheless, the study highlights the need for combined use of questionnaires and spirometry for diagnosis of COPD in future population studies if feasible as self-reported questionnaires alone have a limited sensitivity and specificity for diagnosis. In this largely descriptive analysis, we were unable to identify the sources which explained the variation within and between regions in the prevalence of COPD; the lack of strong associations may reflect lack of specificity in the COPD phenotypes measured, or differences in clinical practice or differences in access to healthcare in China^18^ or to some unmeasured factors responsible for the development of and susceptibility to COPD. Further longitudinal studies are needed to address these questions.

## Conclusions

In summary, the burden of COPD among Chinese adults is high, particularly among those living in rural areas and complicated by a suboptimal management of the disease. The infrequent use of the healthcare system and the low levels of disease awareness reported by those with COPD, therefore, represent a serious deficit in public health services, healthcare education and patient engagement in China which would benefit from additional focused resources.
