# Supplementary material for: Patterns and management of chronic obstructive pulmonary disease in urban and rural China: a community-based survey of 25 000 adults across 10 regions
Source: BMJ Open Respir Res. 2018 Feb 19;5(1):e000267. doi: 10.1136/bmjresp-2017-000267 (PMC5844384; doi:10.1136/bmjresp-2017-000267)
Supplement: Supplementary data [file bmjresp-2017-000267supp001.pdf]

<u>Supplementary data</u>

Patterns and management of chronic obstructive pulmonary disease in urban and rural China: a community-based survey of 25,000 adults across 10 regions

Om P Kurmi PhD, Kourtney Davis PhD, Kin Bong Hubert Lam PhD, Yu Guo MSc, Julien Vaucher MD, Derrick Bennett PhD, Jenny Wang MSc, Zheng Bian MD, Huaidong Du PhD, Liming Li PhD, Robert Clarke FRCP, Zhengming Chen DPhil for the China Kadoorie Biobank Collaborative Group (members listed at the end of paper)

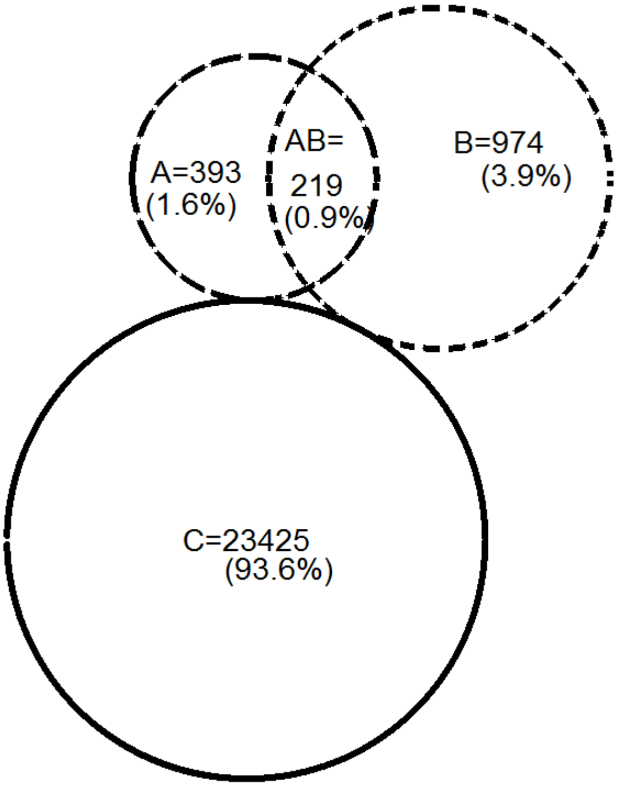

Fig. S1 Venn diagram showing the repartition of participants in the study population.
A= Symptom-based COPD only; B= Doctor-diagnosed COPD only; AB= Symptom-based COPD and Doctor-diagnosed COPD; C=Absence of COPD

Table S1 Prevalence of co-morbidities (self-reported but doctor diagnosed) amongst COPD cases

Characteristics	Study population	Symptom-based COPD[a] % (95% CI)	Doctor-diagnosed COPD[b] % (95% CI)	Symptom-based or doctor-diagnosed COPD[c] % (95% CI)
**All participants (%)**	25011	612 (2.4)	1193 (4.8)	1586 (6.3)
**Co-morbidities**				
Asthma	265	15.7 (14.1, 17.2)	40.9 (38.8, 42.9)	45.7 (43.6, 47.8)
Stroke	853	3.1 (2.6, 3.6)	5.5 (4.8, 6.2)	7.6 (6.8, 8.4)
IHD	1476	4.0 (3.6, 4.5)	8.7 (8.1, 9.3)	10.4 (9.7, 11.1)
Hypertension	6330	2.7 (2.6, 2.9)	5.4 (5.1, 5.6)	7.2 (6.9, 7.4)
Lung cancer	21	2.9 (0.5, 5.3)	6.3 (2.6, 9.9)	9.2 (4.9, 13.5)
Rheumatoid arthritis	1167	4.5 (4.0, 4.9)	8.7 (8.1, 9.3)	11.2 (10.5, 11.9)
Tuberculosis	317	5.3 (4.3, 6.3)	12.7 (11.3, 14.2)	14.0 (12.6, 15.5)
Diabetes	1847	2.4 (2.1, 2.8)	4.2 (3.8, 4.6)	5.9 (5.5, 6.3)

[a]Symptom-based COPD are those with self-reported chronic cough i.e. productive cough for at least 3 months for 2 consecutive years; [b]COPD defined by history of doctor diagnosis of COPD where COPD= COPD and/or Chronic bronchitis and/or emphysema; [c]Either [a] or [b]; Standardised for region, age group and gender as appropriate

Table S2 Functional status impact and management of COPD by region

Characteristics	Total[a]	Rural[b]	Urban[b]
**Participants**	1586	911	675
**CAT-score**			
<10	882 (56.0)	499 (54.8)	383 (56.9)
10−20	514 (33.0)	305 (33.5)	209 (30.9)
>20	190 (11.0)	107 (11.8)	83 (12.2)
**Understanding of condition and treatment**			
Well informed	295 (17.8)	115 (12.6)	180 (26.7)
Inadequately/poorly informed	1114 (71.3)	686 (75.4)	428 (63.4)
Don’t know	177 (10.9)	110 (12.0)	67 (9.9)
**Self-reported severity of chronic lung disease**			
Severe/Very severe	295 (18.7)	192 (21.1)	103 (15.2)
Moderate	578 (36.5)	319 (35.1)	259 (38.4)
Mild/very mild	713 (44.8)	400 (43.9)	313 (46.4)
**Health care professional consultations(last 12 months)**			
No	721 (46.8)	459 (50.3)	262 (38.8)
Yes	865 (53.2)	452 (49.7)	413 (61.2)
General physician	536 (32.7)	294 (32.3)	242 (35.8)
Respiratory specialist	305 (19.1)	141 (15.5)	164 (24.4)
Cardiologist/heart specialist	74 (4.5)	37 (4.1)	37 (5.5)
Traditional Chinese medicine doctor	132 (7.7)	73 (8.0)	59 (8.7)
Other medical professional	104 (6.2)	59 (6.5)	45 (6.6)
**Hospitalisation for COPD (last 12 months)**			
None	1333 (84.6)	765 (84.7)	568 (84.7)
One	182 (12.0)	107 (11.9)	75 (11.2)
≥ Two	58 (3.4)	31 (3.4)	27 (4.1)
**Oxygen therapy at home (last 12 months)**			
No	1523 (96.7)	881 (98.0)	642 (96.1)
Yes	44 (3.3)	18 (2.0)	26 (3.9)
**Vaccination (last 12 months)**			
No	1527 (96.5)	875 (96.1)	652 (96.6)
Yes	59 (3.5)	36 (3.9)	23 (3.4)
Influenza vaccination	54 (2.9)	33 (3.6)	21 (3.1)
Pneumococcal vaccination	14 (1.0)	6 (0.7)	8 (1.2)
**Medication for COPD exacerbation (last 12 months)**			
No	1072 (68.2)	632 (69.4)	440 (65.2)
Yes	514 (31.8)	279 (30.6)	235 (34.8)
Antibiotics	483 (30.2)	264 (29.0)	219 (32.4)
Oral steroids	107 (6.5)	58 (6.4)	49 (7.2)
Injectable steroids	70 (4.6)	30 (3.3)	40 (5.9)
Number of COPD exacerbations after medication			
None	1185 (74.6)	723 (79.4)	462 (68.5)
One	202 (12.9)	80 (8.8)	122 (18.0)
≥ Two	199 (12.6)	108 (11.8)	91 (13.4)
**Prescribed medication (last 7 days)**			
No	1447 (89.4)	840 (92.2)	607 (89.9)
Yes	139 (10.6)	71 (7.8)	68 (10.1)
Short-acting bronchodilators	25 (2.0)	12 (1.3)	13 (1.9)
Long-acting bronchodilators	16 (1.2)	6 (0.7)	10 (1.5)
Oral bronchodilators	19 (1.1)	8 (0.9)	11 (1.6)
Traditional Chinese medicine	33 (2.5)	18 (2.0)	15 (2.2)
Other treatments	94 (7.2)	50 (5.5)	44 (6.6)

[a]Standardised for region, age group and gender; [b] Standardised for age group and gender. Short acting bronchodilators (short-acting beta2-agonist/short-acting muscarinic antagonist); Long-acting bronchodilators (long-acting beta2-agonist / long-acting muscarinic antagonist)

Table S3 Disease severity and management by different COPD criteria by smoking status

Characteristics	Total	Never-regular	Ever-regular
**Participants**	1586	984	602
**CAT-score**			
<10	882 (56.0)	539 (55.7)	343 (56.3)
10−20	514 (33.0)	320 (32.3)	194 (33.7)
>20	190 (11.0)	125 (11.9)	65 (10.0)
**Understanding of condition and treatment**			
Well informed	295 (17.8)	181 (18.8)	114 (16.8)
Inadequately/poorly informed	1114 (71.3)	696 (70.0)	418 (72.6)
Don't know	177 (10.9)	107 (11.2)	70 (10.5)
**Self-reported severity of chronic lung disease**			
Severe/Very severe	295 (18.7)	188 (19.5)	107 (17.9)
Moderate	578 (36.5)	352 (35.3)	226 (37.8)
Mild/very mild	713 (44.8)	444 (45.3)	269 (44.4)
**Health care professional consultations(last 12 months)**			
No	721 (46.8)	416 (42.6)	305 (51.3)
Yes	865 (53.2)	568 (57.4)	297 (48.7)
General physician	536 (32.7)	347 (34.9)	189 (30.4)
Respiratory specialist	305 (19.1)	193 (19.9)	112 (18.3)
Cardiologist/heart specialist	74 (4.5)	45 (4.3)	29 (4.6)
Traditional Chinese medicine doctor	132 (7.7)	96 (9.2)	36 (6.1)
Other medical professional	104 (6.2)	73 (7.0)	31 (5.3)
**Hospitalisation for COPD (last 12 months)**			
None	1333 (84.6)	832 (85.1)	501 (84.0)
One	182 (12.0)	105 (11.1)	77 (12.9)
≥ Two	58 (3.4)	37 (3.7)	21 (3.1)
**Oxygen therapy at home (last 12 months)**			
No	1523 (96.7)	948 (97.3)	575 (96.1)
Yes	44 (3.3)	24 (2.7)	20 (3.9)
**Vaccination (last 12 months)**			
No	1527 (96.5)	946 (96.5)	581 (96.6)
Yes	59 (3.5)	38 (3.5)	21 (3.4)
Influenza vaccination	54 (2.9)	35 (3.2)	19 (2.6)
Pneumococcal vaccination	14 (1.0)	10 (0.9)	4 (1.1)
**Medication for COPD exacerbation (last 12 months)**			
No	1072 (68.2)	656 (68.0)	416 (68.3)
Yes	514 (31.8)	328 (32.0)	186 (31.7)
Antibiotics	483 (30.2)	308 (30.1)	175 (30.2)
Oral steroids	107 (6.5)	64 (5.8)	43 (7.3)
Injectable steroids	70 (4.6)	44 (3.8)	26 (5.4)
Number of COPD exacerbations after medication			
None	1185 (74.6)	724 (75.1)	461 (74.0)
One	202 (12.9)	134 (12.7)	68 (13.1)
≥ Two	199 (12.6)	126 (12.2)	73 (12.9)
**Prescribed medication (last 7 days)**			
No	1447 (89.4)	903 (91.0)	544 (87.7)
Yes	139 (10.6)	81 (9.0)	58 (12.3)
Short-acting bronchodilators	25 (2.0)	12 (1.3)	13 (2.7)
Long-acting bronchodilators	16 (1.2)	9 (0.9)	7 (1.5)
Oral bronchodilators	19 (1.1)	11 (1.1)	8 (1.1)
Traditional Chinese medicine	33 (2.5)	22 (2.5)	11 (2.5)
Other treatments	94 (7.2)	55 (6.2)	39 (8.2)

All the figures are standardised for region, age group and gender. Short acting bronchodilators (short-acting beta2-agonist/short-acting muscarinic antagonist); Long-acting bronchodilators (long-acting beta2-agonist / long-acting muscarinic antagonist)

Table S4 Disease severity and management measures, by COPD sub-types (COPD LLN)

Characteristics	Study population	Symptom-based COPD % (95% CI)	Doctor-diagnosed COPD % (95% CI)	Symptom-based or doctor-diagnosed COPD % (95% CI)
**Participants**	600	209	490	600
**Lung function (mean ± SD)**				
FEV1 (L)	1.4 ± 0.1	1.5 ± 0.1	1.3 ± 0.1	1.4 ± 0.1
FVC (L)	2.4 ± 0.2	2.6 ± 0.2	2.3 ± 0.2	2.4 ± 0.2
FEV1/FVC (%)	56.4 ± 2.0	58.0 ± 2.0	55.2 ± 2.0	56.4 ± 2.0
**CAT-score**				
<10	311	45.4 (43.1, 47.8)	50.5 (49.0, 52.1)	51.8 (50.5, 53.2)
10−20	204	34.1 (31.9, 36.3)	34.7 (33.2, 36.2)	34.0 (32.7, 35.3)
>20	85	20.4 (18.5, 22.4)	14.8 (13.7, 15.8)	14.2 (13.2, 15.1)
**Awareness of condition and treatment**				
Well informed	97	12.7 (11.1, 14.3)	17.7 (16.6, 18.9)	16.2 (15.2, 17.2)
Inadequately/poorly informed	438	74.6 (72.6, 76.7)	72.5 (71.2, 73.9)	73.0 (71.8, 74.2)
Don't know	65	12.7 (11.2, 14.2)	9.7 (8.8, 10.7)	10.8 (10.0, 11.7)
**Self-reported severity of chronic lung disease**				
Severe	150	26.7 (24.6, 28.8)	27.3 (25.9, 28.7)	25.0 (23.8, 26.2)
Moderate	229	36.7 (34.4, 38.9)	39.4 (37.9, 40.9)	38.2 (36.8, 39.5)
Mild	221	36.6 (34.4, 38.8)	33.3 (31.9, 34.7)	36.8 (35.5, 38.2)
**Vaccination (last 12 months)**				
No	580	96.9 (96.1, 97.7)	96.1 (95.5, 96.7)	96.7 (96.2, 97.2)
Yes	20	3.1 (2.3, 3.9)	3.9 (3.3, 4.5)	3.3 (2.8, 3.8)
Influenza vaccination	18	2.2 (1.5, 2.8)	3.7 (3.1, 4.3)	3.0 (2.5, 3.5)
Pneumococcal vaccination	3	1.0 (0.5, 1.4)	0.4 (0.2, 0.7)	0.5 (0.3, 0.7)
**Health care professional consultations(last 12 months)**				
No	259	52.1 (49.8, 54.4)	36.1 (34.7, 37.6)	43.2 (41.8, 44.5)
Yes	341	47.9 (45.6, 50.2)	63.9 (62.4, 65.3)	56.8 (55.5, 58.2)
General physician	216	31.5 (29.3, 33.6)	40.3 (38.8, 41.8)	36.0 (34.7, 37.3)
Respiratory specialist	126	18.1 (16.3, 19.9)	23.7 (22.4, 24.9)	21.0 (19.9, 22.1)
Cardiologist/heart specialist	33	3.6 (2.7, 4.6)	6.6 (5.9, 7.4)	5.5 (4.9, 6.1)
Traditional Chinese medicine doctor	49	5.8 (4.7, 6.9)	9.6 (8.7, 10.4)	8.2 (7.4, 8.9)
Other medical professional	31	3.5 (2.6, 4.3)	5.7 (5.0, 6.5)	5.2 (4.6, 5.8)
**Hospitalisation for COPD (last 12 months)**				
None	481	84.5 (82.8, 86.3)	78.4 (77.1, 79.6)	81.3 (80.2, 82.3)
One	84	10.8 (9.3, 12.3)	16.4 (15.2, 17.5)	14.2 (13.2, 15.1)
≥ Two	27	4.7 (3.6, 5.7)	5.3 (4.6, 5.9)	4.6 (4.0, 5.1)
**Oxygen therapy at home (last 12 months)**				
Yes	26	4.0 (3.1, 4.9)	5.1 (4.4, 5.8)	4.4 (3.8, 5.0)
No	563	96.0 (95.1, 96.9)	94.9 (94.2, 95.6)	95.6 (95.0, 96.2)
**Medication for COPD exacerbation (last 12 months)**				
No	390	69.1 (67.0, 71.3)	60.8 (59.4, 62.3)	65.0 (63.7, 66.3)
Yes	210	30.9 (28.7, 33.0)	39.2 (37.7, 40.6)	35.0 (33.7, 36.3)
Antibiotics	196	28.5 (26.4, 30.6)	36.9 (35.5, 38.3)	32.7 (31.4, 33.9)
Oral steroids	56	8.9 (7.6, 10.2)	10.5 (9.6, 11.4)	9.3 (8.5, 10.1)
Injectable steroids	25	2.9 (2.1, 3.7)	4.8 (4.2, 5.5)	4.2 (3.6, 4.7)
Number of COPD exacerbations after medication				
None	440	75.7 (73.7, 77.7)	69.4 (68.0, 70.7)	73.3 (72.1, 74.5)
One	68	6.4 (5.3, 7.6)	13.7 (12.6, 14.7)	11.3 (10.5, 12.2)
≥ Two	92	17.9 (16.1, 19.7)	17.0 (15.8, 18.1)	15.3 (14.3, 16.3)
**Prescribed medication (last 7 days)**				
No	529	90.0 (88.6, 91.5)	86.2 (85.2, 87.3)	88.2 (87.3, 89.1)
Yes	71	10.0 (8.5, 11.4)	13.8 (12.7, 14.8)	11.8 (10.9, 12.7)
Short-acting bronchodilators	13	2.5 (1.8, 3.2)	2.4 (2.0, 2.9)	2.2 (1.8, 2.6)
Long-acting bronchodilators	9	2.0 (1.3, 2.7)	1.6 (1.2, 2.0)	1.5 (1.2, 1.8)
Oral bronchodilators	10	1.0 (0.5, 1.5)	2.0 (1.6, 2.4)	1.7 (1.3, 2.0)
Any inhalers	8	0.5 (0.2, 0.9)	1.4 (1.1, 1.8)	1.3 (1.0, 1.6)
Traditional Chinese medicine	17	2.0 (1.4, 2.7)	3.4 (2.9, 4.0)	2.8 (2.4, 3.3)
Other treatments	44	6.3 (5.2, 7.5)	8.4 (7.5, 9.2)	7.3 (6.6, 8.1)

All data standardised for region, age group and gender as appropriate. Short acting bronchodilators (short-acting beta2-agonist/short-acting muscarinic antagonist); Long-acting bronchodilators (long-acting beta2-agonist / long-acting muscarinic antagonist)

Table S5 Use of medication among COPD cases who reported taking medication in the last 7 days or in the last 12 months prior to re-survey by gender, region, socioeconomic and smoking status (COPD LLN)

Characteristics	Study population	Use of medication (%, 95% CI) Last 7 days[a]	Use of medication (%, 95% CI) Last 12 months[b]
**Participants**	600	71	210
**Sex**			
Men	325	12.6 (10.9, 14.4)	34.5 (32.1, 37.0)
Women	275	10.8 (9.0, 12.6)	35.6 (32.8, 38.3)
**Area**			
Rural	414	9.9 (8.5, 11.4)	30.6 (28.4, 32.8)
Urban	186	16.2 (13.6, 18.9)	44.6 (41.0, 48.1)
**Regions[c]**			
Sichuan (R)	138	9.5 (6.9, 12.1)	9.4 (6.9, 12.0)
Gansu (R)	73	12.5 (8.6, 16.3)	68.5 (63.2, 73.8)
Zhejiang (R)	77	7.3 (4.4, 10.1)	25.1 (20.2, 29.9)
Harbin (U)	47	17.7 (12.3, 23.1)	48.8 (41.4, 56.3)
Henan (R)	49	0.0 (0.0, 0.0)	15.9 (10.4, 21.4)
Qingdao (U)	35	13.6 (8.1, 19.2)	42.2 (34.7, 49.7)
Hunan (R)	77	16.4 (12.1, 20.6)	47.3 (41.8, 52.8)
Liuzhou (U)	39	26.7 (19.8, 33.5)	36.5 (28.8, 44.2)
Haikou (U)	17	20.5 (11.8, 29.2)	57.7 (44.9, 70.4)
Suzhou (U)	48	6.5 (3.0, 9.9)	46.3 (39.3, 53.2)
**Household income (Yuan)**			
<10000	86	9.3 (7.3, 11.4)	35.4 (32.0, 38.8)
10000 - 19999	82	17.4 (14.8, 19.9)	44.2 (40.8, 47.5)
≥ 20000	432	11.4 (10.4, 12.5)	34.0 (32.5, 35.4)
**Education**			
None	140	8.5 (6.8, 10.1)	32.6 (29.6, 35.5)
Primary	246	13.2 (11.6, 14.8)	38.5 (36.5, 40.5)
Secondary or tertiary	214	11.8 (10.3, 13.4)	29.9 (27.7, 32.1)
**Smoking status**			
Never	304	13.1 (11.5, 14.6)	35.6 (33.5, 37.7)
Ex-regular	92	14.6 (11.9, 17.2)	39.0 (35.8, 42.3)
Current regular	204	10.9 (9.0, 12.8)	29.9 (27.3, 32.5)

[a]Medication questionnaire was completed only by those who had symptom-based COPD or history of doctor diagnosed COPD in the 2nd resurveys; Medication in last 7 days (Short-acting bronchodilators, long-acting bronchodilators, oral bronchodilators, any inhaled medications, traditional Chinese medicine and any other treatments prescribed for COPD);
[b]Medication in last 12 months (Antibiotics, oral corticosteroids, injectable corticosteroids). Standardised for region, age group and gender as appropriate;
[c]The regions are ordered by COPD prevalence as in Figure 3.

Appendix 1: COPD questionnaire (Q7.5)

The following questions are related to your chronic lung disease. Please give the answers you feel most appropriate.

1. **Overall, how would you rate the severity of your chronic lung disease now?**

   ☐ Very severe
   ☐ Severe
   ☐ Moderate
   ☐ Mild
   ☐ Very mild

2. **How does your doctor classify the severity of your condition?**

   ☐ Stage1 (mild)
   ☐ Stage 2 (moderate)
   ☐ Stage 3 (severe)
   ☐ Stage 4 (very severe)
   ☐ Doctor has not diagnosed or told me
   ☐ Don't know

3. **The next part of the questionnaire is to measure the impact of respiratory symptoms on wellbeing and your daily life. Please indicate on a scale of 0 to 5 how strongly you feel about the two pair of the statements related to each question.**

	Score					
	**0**	**1**	**2**	**3**	**4**	**5**
	☐	☐	☐	☐	☐	☐

1) **Frequency of your cough?**
   0= Never cough; 5= Cough all the time

2) **Amount of mucus in your chest?**
   0= No mucus at all; 5= Full of mucus

3) **Tightness of your chest?**
   0= Not tight at all; 5= Very tight

4) **Breathless when walking up a hill/one flight of stairs?**
   0= Not breathless; 5= Very breathless

5) **Limitation of daily activities at home by the condition?**
   0= Not limited; 5= Very limited

6) **Degree of confidence about leaving your home?**
   0= Confident; 5= Not confident at all

7) **Quality of sleep at night and whether it is affected by the condition?**

0= I sleep soundly; 5= I don't sleep soundly because of my lung condition

8) **Usual levels of energy?**
0= I have lots of energy; 5= I have no energy at all

4. Have you ever had your lung function tested by blowing hard into a tube (excluding the present and previous CKB tests)?

- ☐ Yes
- ☐ No
- ☐ Don't know

5. Have you ever had your lung function tested in a cabin, called a phlethysmograph?

- ☐ Yes
- ☐ No

6. Don't know**In the past 12 months what types of health care professionals have you seen about your condition?**

Yes	No	
☐	☐	General physician
☐	☐	Respiratory specialist
☐	☐	Cardiologist/heart specialist
☐	☐	Traditional Chinese medicine doctor
☐	☐	Local health centre or village doctor
☐	☐	Other medical professional

7. In the past 12 months, how many times have you been hospitalised overnight, or longer, as a direct result of your condition? _____ times (If none, put 0)

8. In the past 12 months, have you taken any of the following to treat worsening of your breathing problems?

Yes	No	
☐	☐	Antibiotics
☐	☐	Oral steroids
☐	☐	Injectable steroids

} → ***If ticked "NO" for all items, Go to <u>Q9.</u>***

8.1 In the past 12 months, how many episodes of COPD exacerbation have you had when you took antibiotics, oral steroids, or injectable steroids to treat worsening of your breathing problems? (if none, put 0) _____ times

9. In the past seven days, have you taken any prescriptive medicine (such as

inhalers, tablets or something else) for the condition?

- ☐ Yes → ***If ticked, Go to <u>Q9.1</u>***
- ☐ No
- ☐ Don't know

9.1 What types of medication have you taken in the past seven days?

Yes	No	
☐	☐	Short-acting beta-agonists (e.g. albuterol, salbutamol)
☐	☐	Short-acting anti-muscarinic agents (e.g. Atrovent, Combivent
☐	☐	Long-acting beta-agonists (salmeterol/Serevent, formoterol/Foradil)
☐	☐	Long-acting anti-muscarinic agent (tiotropium/Spiriva)
☐	☐	Oral bronchodilators
☐	☐	Long-acting beta agonist+inhaled corticosteroid combination inhalers
☐	☐	Inhaled corticosteroids
☐	☐	Traditional Chinese medicine
☐	☐	Other treatments

10. In the past 12 months have you used home oxygen for your condition?

- ☐ Yes → ***If ticked, Go to <u>Q10.1</u>***
- ☐ No
- ☐ Don't know

10.1 About how often do you use oxygen at home?

- ☐ Every day
- ☐ Most days
- ☐ Once a week
- ☐ Once a month
- ☐ Only occasionally

11. In the past 12 months, have you had an influenza vaccine (also called a flu shot)?

- ☐ Yes
- ☐ No
- ☐ Don't know

12. In the past 12 months, have you had a vaccine or shot which protects you against pneumonia?

- ☐ Yes
- ☐ No
- ☐ Don’t know

13. How well informed do you feel you are about your condition and its treatment?

- ☐ Well informed
- ☐ Less than adequately informed
- ☐ Very poorly informed
- ☐ Don’t know
